# Design of Resistor-Capacitor Physically Unclonable Function for Resource-Constrained IoT Devices †

**DOI:** 10.3390/s20020404

**Published:** 2020-01-10

**Authors:** Sangjae Lee, Mi-Kyung Oh, Yousung Kang, Dooho Choi

**Affiliations:** Electronics and Telecommunications Research Institute, Daejeon 34129, Korea; ohmik@etri.re.kr (M.-K.O.); youskang@etri.re.kr (Y.K.); dhchoi@etri.re.kr (D.C.)

**Keywords:** physically unclonable function, hardware security, passive component, resistor-capacitor, IoT

## Abstract

Keeping IoT devices secure has been a major challenge recently. One of the possible solutions to secure IoT devices is to use a physically unclonable function (PUF). A PUF is a security primitive that can generate device-specific cryptographic information by extracting the features of hardware uncertainty. Because PUF instances are very difficult to replicate even by the manufacturer, the generated bit sequence can be used as cryptographic keys or as a unique identifier for the device. Regarding the implementation of PUF, the majority of PUFs introduced over the past decade are in the form of active components and have been implemented as separate chips or embedded as a part of a chip, making it difficult to use them in low-cost IoT devices due to cost and design flexibility. One approach to easily adopt PUFs in resource-constrained IoT devices is to use passive components such as resistors and capacitors (RC) that can be configured at low cost. The main feature of this RC-based PUF is that it extracts the small difference caused by charging and discharging of RC circuits and uses it as a response. In this paper, we extend the previous research and show the possibility to secure IoT devices by using the RC-based PUF.

## 1. Introduction

Recently, as the amount of IoT devices has increased tremendously, there has been considerable interest in the security issues of IoT devices. Due to the limited resources of IoT devices, it is difficult to install the conventional security solutions [1]. In addition, one of the biggest weakness in IoT device security is that many of them come set up with a default ID/PW. It has been shown that just a small set of ID/PW gives an access to a great number of IoT devices. Based on this vulnerability, the largest Distributed Denial-of-Service (DDoS) attack ever was launched using an IoT botnet (Mirai Botnet) in October of 2016. Therefore, it is highly required to ensure that IoT devices are autonomously secured, where ID/PW or security keys are set and managed without user intervention. 

One approach to enhance the security of IoT devices is to utilize physically unclonable functions (PUFs). A PUF has emerged as a new alternative to meet the increasing demand for protection of embedded systems against security attacks such as replication of semiconductors, or stealing default ID/PW or security keys stored in nonvolatile memory. It generates device-specific random sequences unique to a semiconductor or hardware by using the innate hardware uncertainty derived from the manufacturing process [2,3,4].

Since a PUF is extremely difficult to replicate even by the manufacturer, it can be used to generate and maintain secret credentials that are even unknown to semiconductor architectures or device programmers. In the process of creating and maintaining a secret key or ID/PW unique to a device, it does not store the secret information in the non-volatile memory (NVM). Therefore, even if one device is attacked, the same attack vector may not be used for other devices, which makes a PUF as a promising primitive for IoT devices to prevent large-scale DDoS attacks [5]. Exploiting the PUF as a source of “secret” information, it can be used in applications such as secret key management, data privacy, device authentication, and intellectual property (IP) protection of FPGAs or chips [6].

There are various works related with PUFs, including delay-based PUF [1,6,7,8,9], and memory-based PUF [10,11] over the past decade. Please refer to [6] for a comprehensive overview on PUFs. In terms of implementation methods, the majority of such PUFs must be fabricated as separate ASICs or FPGAs, or embedded in the chip at the design time. In recent years, there have been attempts to implement a PUF using the components of the device itself such as dynamic RAM (DRAM) PUF [12,13] and radio frequency (RF) PUF [14]. DRAM PUFs identified unexpected setup behavior in commercial off-the-shelf DRAMs that could be used as a PUF. RF PUFs identified the effect of variations in analog, RF, and mixed signal properties in wireless radios, where inherent RF properties were used to make a PUF without any additional hardware. Some studies have shown that it is possible to construct SRAM PUFs using existing off-the-shelf components [15]. The advantage of these PUFs is that they can be implemented without any extra hardware.

In terms of circuit for implementing a PUF, another interesting approach is to use analog circuits as a PUF primitive [16]. This study showed an analog PUF structure based on analog and mixed signal circuits. In this implementation, an input challenge was converted to an analog signal by digital-to-analog converters (DACs) and passed through low pass filters and amplifiers. The resulting signal was sampled by an analog-to-digital converter (ADC), and a response was generated from sampled bits.

Recently, a resistor-capacitor (RC) PUF has been proposed that used passive components such as resistors and capacitors as a PUF primitive [17]. Focusing on the fact that resistors and capacitors have inherent tolerances that arise in the manufacturing process, it achieved PUF-like properties by combining those components with ADCs embedded in many commercial microcontrollers (MCUs). The RC PUF adopts analog circuits and an ADC as PUF primitives, which is similar to [16]. However, instead of exploiting active components such as amplifiers, only passive components are simply employed for analog circuits and DACs are not used. The motivation for this work is similar with [14] in that it can provide affordable PUF functionality for resource-constrained IoT devices without using previously mentioned on-chip/off-chip PUF-dedicated hardware.

In this paper, we extend our previous work published in [17]. In the previous work, only first-order RC circuit was used for the RC PUF. In this work, we add a second-order RC circuit to evaluate the performance change due to the increase in RC components and circuit types. In addition, we provide randomness characteristics of the RC PUF with the NIST statistical test suite instead of uniformity used in the previous work. We explain the operation and the architecture of the RC PUF again, describing how to choose resistor and capacitor components and other parameters. Finally, we present experimental results to characterize the RC PUF with two types of RC circuits.

## 2. RC PUF Design

### 2.1. RC PUF Architecture

**Figure 1:**
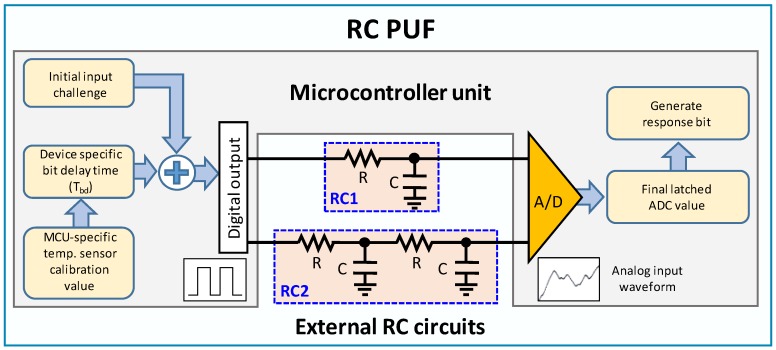
Hardware and software block diagram of the resistors and capacitors physically unclonable function (RC PUF).

The basic structure of the proposed RC PUF is shown in Figure 1, where the second-order RC circuit (RC2) is newly added to the original RC PUF in [17]. Two types of RC circuits are the simplest form commonly used and were chosen for ease of implementation. Although new circuit has been added, the operation of the proposed RC PUF is same as the original RC PUF in that the RC PUF generates 1-bit response for a challenge consisting of multiple bits. The operation of the RC PUF is following as explained in [17]. At first, the input challenge, which consists of multiple bits, is shifted by 1 bit and the corresponding bit is applied sequentially to the digital output pin such as a general purpose I/O (GPIO). Assuming that the I/O voltage of the MCU is 3.3 V, the voltage applied to the input of the RC circuit will be 3.3 V or ground (GND or 0 V) depending on the value of the output bit. The GPIO output or the input of the RC circuit (RCin) remains constant for a period of time for one bit, which is defined as “bit delay time.” After the bit delay time for one bit has expired, the output changes by the next bit. Accordingly, the output of the RC circuit gradually increases (charging) or decreases (discharging). After all the bits of one challenge bit stream are passed to the GPIO, the final output voltage of the RC circuit (RCout) is sampled by the ADC, and 1-bit response is extracted from the ADC value. We use different GPIO output and ADC input pins for two types of the RC circuit, and only one circuit operates at a time during one challenge sequence so that one circuit does not affect the operation of the other.

In order to utilize the RC circuit as a PUF primitive, the input voltage of the RC circuit must be changed during a transient-state before the output of the RC circuit by a previous bit is fully charged (3.3 V) or discharged (GND), i.e., a steady-state. The change time of the input of the RC circuit is determined by the constant bit delay time depicted in Figure 1. The bit delay time should not be too long so that the RC circuit does not enter a steady-state. If not, the output of the RC circuit will be stuck at 3.3 V or GND for all input challenges. In a steady-state, the effect of component tolerance almost disappears, and it is not easy to differentiate response values between different challenges or devices. Furthermore, it is important to keep the bit delay time as small as possible within the transient-state because the longer the bit delay time is, the longer the response generation time in the RC PUF will be, which negatively affects applications that use the PUF. This bit delay time can be adjusted easily from 1 microsecond to tens of microseconds using the software delay function on MCUs.

Since the proposed RC PUF is composed of RC circuits and one MCU, the unique characteristics of MCU can be used to generate a device-specific bit delay time. In order to compare the performance differences of the RC PUF due to the device-specific bit delay time for each device, we consider two approaches. One is to use the initial fixed bit delay time for all RC PUF instances. The other is to use its unique bit delay time for each RC PUF, where the temperature sensor calibration value is added to the initial fixed bit delay time to generate its own unique bit delay time, as shown in Figure 1. The MCU used in the RC PUF stores the temperature sensor calibration values generated in the manufacturing process.

We note that the bit delay time is physically related to the product of the resistance and the capacitance, i.e., *τ* = *RC* [18], where *R* is the resistance of the resistor, and *C* is the capacitance of the capacitor. Therefore, the resistance and capacitance must be selected considering *τ* close to the bit delay time. When selecting the capacitance, both probing analysis and reliability should be considered. Generally, the input capacitance of general oscilloscope probes ranges up to 100 pF [19]. If the capacitance of the RC circuit is much greater (e.g., 1 uF) than the input capacitance of the probe, probing has little effect on the rise and fall time of the RC circuit. In this case, the correct ADC voltage can be read by probing, making the RC PUF vulnerable to probing analysis. On the contrary, if the capacitance is too small (e.g., less than a few tens of pF), probing will increase the total capacitance of the RC circuit, making it difficult to estimate the original ADC value. Although a small capacitance of the RC circuit is recommended to prevent probing analysis, experiments with the RC PUF have shown that reliability is more severely affected if the capacitance is less than a few tens of pF. As a result, the capacitance of the RC circuit is chosen between 100 pF and 200 pF to ensure reliability while preventing probing analysis to some extent. Since the capacitance is determined, the resistance is set to 100 kΩ so that the bit delay time can be up to tens of microseconds.

Next, after the resistance and capacitance have been fixed, the appropriate type of resistor and capacitor for the RC PUF should be selected. The main concern when selecting RC components is to choose them so that the tolerance must be as large as possible to differentiate the response correlation between different PUFs, while providing robust properties against temperature and voltage variations and aging.

In the proposed RC PUF, we exploit commonly used thin film resistors and multilayer ceramic capacitors (MLCCs) for the RC circuit. We use class 1 (C0G) capacitors with a 20% tolerance for the capacitor of RC circuits. Class 1 capacitors are generally more robust to temperature variation and aging than class 2 or class 3 capacitors [20,21,22]. Then, we choose a resistor with a tolerance of 1% and a good temperature coefficient of 25 ppm for the RC circuit [23]. The selected resistor and capacitor can be easily purchased for less than a few cents. Therefore, the RC PUF can be implemented at a lower cost than other PUFs implemented as separate chips or built into SoCs. In addition, the RC circuit is separate from the microcontroller, providing design flexibility because there is no need to use a specific chip with a built-in PUF.

### 2.2. RC PUF Simulation

Although the simulation on the “transient-state” characteristics at the RC circuit output is depicted in [17], we briefly describe again about the the behavior of the first-order RC circuit (RC1), as shown in Figure 1. The basic representation of a first-order RC circuit is divided into two modes—rising (or charging) and falling (or discharging). The voltage changes at the time of charging and discharging the RC1 can be represented as [18]

(1) $$V\left(t\right)=V_{max}\left(1-e^{-\frac{t}{RC}}\right),\;\mathrm{for}\;\mathrm{charging},$$

(2) $$V\left(t\right)=V_{0}e^{-\frac{t}{RC}},\;\mathrm{for}\;\mathrm{discharging},$$

where $V_{max}$ is the maximum voltage applied to the input of the RC circuit, and $V_{0}$ is the initial input voltage of the RC circuit at the start of discharging.

**Figure 2:**
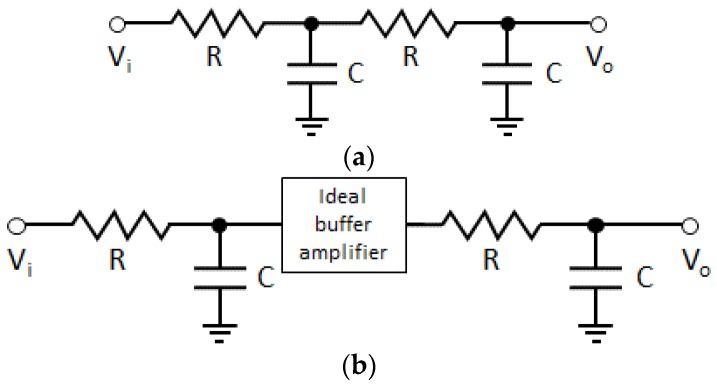
Second-order RC circuit used in RC PUF (**a**) and with ideal buffer amplifier (**b**).

As we mentioned earlier, it is necessary to calculate the voltage changes in the “transient-state” during the RC PUF operation. To do this, we modify (1) and (2) to indicate charge and discharge behavior starting at the last voltage value of the previous “transient-state” when a new input value is applied to the RC circuit. Thus, the modified voltage can be expressed as

(3) $$V\left(t+1\right)=V\left(t\right)+V_{rise}\left(1-e^{-\frac{T_{bd}}{RC}}\right),\;\mathrm{for}\;\mathrm{charging},$$

(4) $$V\left(t+1\right)=V\left(t\right)e^{-\frac{T_{bd}}{RC}},\;\mathrm{for}\;\mathrm{discharging},$$

where the difference between $V_{max}$ and the voltage V(t+1)  at the start time of charging is $V_{rise}=V_{max}-V(t$), and $T_{bd}$ is the bit delay time. As shown in (3) and (4), the voltage changes up to the next time are affected by the previous voltage V(t). Figure 2 shows an example where bit values of ‘101’ are sequentially applied to the input of the RC circuit, where the initial RC input voltage is $V_{0}=\;$0 V, and $V_{max}=3.3\;V$. The voltage change for the first bit ‘1′ after $T_{bd}$ is then calculated as follows according to (3).

(5) $$V_{1}=V_{max}\left(1-e^{-\frac{T_{bd}}{RC}}\right).$$

If the second bit ‘0′ is applied to the RC input, the voltage change according to (4) is then written as

(6) $$V_{2}=V_{1}e^{-\frac{T_{bd}}{RC}}.$$

Next, for the third bit ‘1′, the voltage change is calculated by using (3) and (6), which can be given by

(7) $$\begin{array}{l} V_{3}=V_{2}+\left(V_{max}-V_{2}\right)\left(1-e^{-\frac{T_{bd}}{RC}}\right)\\ =V_{1}e^{-\frac{T_{bd}}{RC}}+\left(V_{max}-V_{1}e^{-\frac{T_{bd}}{RC}}\right)\left(1-e^{-\frac{T_{bd}}{RC}}\right), \end{array}$$

where $V_{1}$ is calculated in (5). It is observed from (6) and (7) that the voltage change due to the sequence of bit patterns accumulates the influence of the previous values.

Next, compared to the first-order RC circuit, the representation of time domain response of the second-order RC circuit (RC2) is more complicated. Time domain responses for complex circuits can usually be found by using the Laplace transform to determine the transfer function for the circuit and by taking an inverse Laplace transform. For the RC2 as shown in Figure 2a, the transfer function can be expressed as [24]

(8) $$\frac{V_{o}}{V_{i}}=\frac{w_{n}{}^{2}}{s^{2}+3w_{n}s+w_{n}{}^{2}}=\;\frac{w_{n}{}^{2}}{s^{2}+2\zeta w_{n}s+w_{n}{}^{2}},$$

where $w_{n}=1/RC$ and ζ=3/2. Since ζ>1, the RC2 can be considered as an overdamped case, and the time domain response at the time of charging can be obtained by taking inverse Laplace transform of the product of the unit step input signal (*1*/*s*) and the transfer function (8), which can be given by [25]

(9) $$\begin{array}{c} \frac{V_{o}\left(t\right)}{V_{i}\left(t\right)}={ℒ}^{-1}\frac{1}{s}\;\frac{w_{n}{}^{2}}{s^{2}+2\zeta w_{n}s+\;w_{n}{}^{2}}\\ =1+\frac{\left(\zeta -\sqrt{\zeta^{2}-1}\right)e^{-w_{n}\left(\zeta +\sqrt{\zeta^{2}-1}\right)t}-\left(\zeta +\sqrt{\zeta^{2}-1}\right)e^{-w_{n}\left(\zeta -\sqrt{\zeta^{2}-1}\right)t}}{2\sqrt{\zeta^{2}-1}}. \end{array}$$

Although (9) represents the exact time response for the second order RC circuit at the time of charging, it is difficult to figure out the effect of the additional RC elements when compared to (1) of the RC1. To make it easier to understand the effect of the additional RC elements, a second-order RC circuit with two first-order RC circuits connected by an ideal buffer amplifier can be considered as shown in Figure 2b although slightly different from the RC2 used in the RC PUF [26]. In this case, the voltage changes at the time of charging the RC2 can be represented as

(10) $$\frac{V_{o}\left(t\right)}{V_{i}\left(t\right)}=1-e^{-\frac{t}{RC}}-\frac{t}{RC}e^{-\frac{t}{RC}}.$$

Although this expression differs from (9) and does not represent the exact behavior of RC2, it shows that the rise time at the time of charging the RC2 is slower than that of RC1 due to the final term in (10). In addition, the voltage changes at the time of discharging RC2 can be inferred in a similar way with reference to the above equation, which can be written as

(11) $$\frac{V_{o}\left(t\right)}{V_{i}\left(t\right)}=e^{-\frac{t}{RC}}+\frac{t}{RC}e^{-\frac{t}{RC}}.$$

As can be seen in (11), the falling time at the time of discharging the RC2 is also slower than that of RC1 due to the second term in (11) when compared to (2) of the RC1.

**Figure 3:**
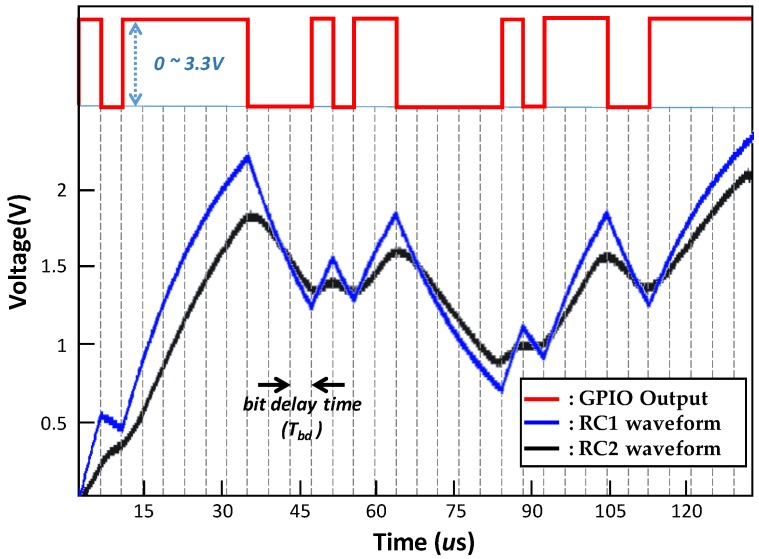
Comparison of simulation and experimental waveforms.

Figure 3 shows the experiment waveforms for the first-order RC circuit (RC1) and the second-order RC circuit (RC2). The red line represents the digital (GPIO) output waveform of the RC PUF to run both RC1 and RC2. We applied an arbitrary 32-bit challenge shown at the top of the figure. The resistance and capacitance of the RC circuit are *R* = 100 kΩ and *C* = 200 pF, respectively. The maximum input voltage to the RC circuit is $V_{max}$ = 3.3 V and the bit delay time is about 4.3 us.

As expected, the output of the RC circuit changes according to the challenge bit pattern during the “transient-state.” In the ideal case, the output value of the RC circuit according to the same bit pattern should be the same for different RC PUFs. However, the component tolerance, i.e., *R* and *C*, causes a difference in the sampled ADC value between different RC PUFs. For this reason, as explained in [17], we can see in that the blue line of experimental waveform for RC1 differs from the red line of simulation waveform for the first-order RC circuit. In addition, because the time constant of RC2 is greater than that of RC1, the black line of experimental waveform for RC2 is significantly different from RC1 as can be seen in Figure 3. In conclusion, the tolerance of these RC components causes the responses of the RC PUF to be uncorrelated with each other.

### 2.3. RC PUF Implementation

**Figure 4:**
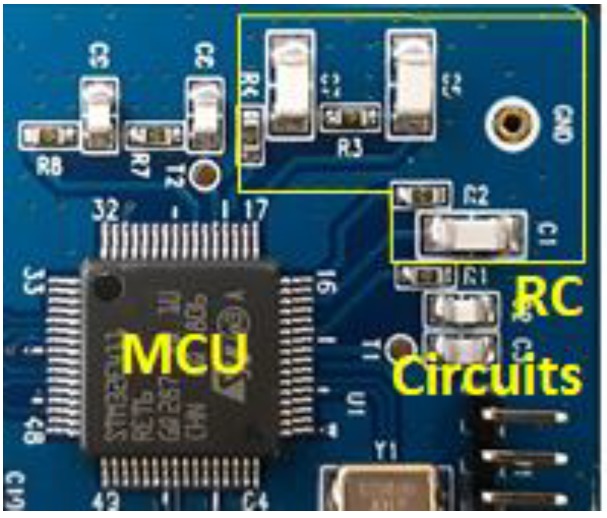
RC circuits and microcontroller (MCU) on the RC PUF board.

We implemented and tested the RC PUF board equipped with two different RC circuits and one MCU, as shown in Figure 4. The MCU equipped on the board is a STM32F4 series MCU from STMicroelectronics, which has a built-in ADC and stores the temperature sensor calibration value as mentioned previously [27]. The supply voltage of the MCU is 3.3 V. Hence, the output voltage of the GPIO used as the input of the RC circuit is also 3.3 V. We also constructed a testbed using 100 RC PUF boards to measure the performance of the RC PUF.

As mentioned earlier, we consider two types of RC circuits to understand the effects of different types of RC circuits on RC PUF behavior. One is a first-order RC circuit (RC1) with one 100 kΩ resistor and one 200 pF capacitor. The other is a second-order RC circuit (RC2) consisting of two resistors and two capacitors with a resistance and a capacitance of 100 kΩ and 100 pF, respectively. The resistance and capacitance of RC1 are the same as those used in the simulation.

In order to compare the simulation results with the experimental results, we run the RC PUF for both RC1 and RC2 using the same challenge used in the simulation. As shown in Figure 3, the experimental waveform of the RC PUF using RC1 is similar to the simulation waveform, and the implemented RC PUF works as expected when compared to the simulation. The difference between the simulation and the experimental waveform of RC1 is considered to be caused by the component tolerance of *RC* and other on-board components. The rise and fall times of RC2 are greater than RC1, leading to different waveforms from RC1. The difference in performance between two types will be described in detail in Section 3.

**Figure 5:**
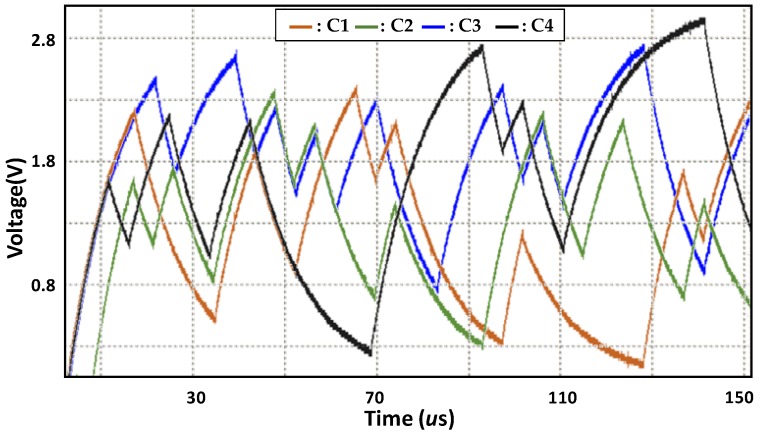
Experimental waveforms of RC1 for different challenges.

Finally, we compare RC PUF waveforms for different challenges. We run the RC PUF using RC1 on one RC PUF board for four randomly selected 32-bit challenges (C1~C4), as shown in Figure 5. It is observed that different challenge patterns exhibit different waveforms, resulting in a significant difference in the sampled ADC value.

## 3. Experimental Results

As described in Section 2, once all bits of one challenge are applied to the RC circuit, the final output value of the RC circuit is sampled by the ADC. Then the sampled ADC value is used to generate a response bit. Since the ADCs built into the MCU typically support multiple bit resolution, e.g., 12-bit, we need to determine which bit is used for the response bit among multiple bits. For example, the valid bit must be selected from bit 11 to bit 0, where bit 0 represents the least significant bit.

The response bit between different PUFs for the same challenge is more likely to be the same as the selected bit position is closer to the most significant fit. For example, the experiment using the RC PUF shows that the differences in ADC sampling values in different PUFs for the same challenge are in the order of tens to hundreds, which means that the response bit can be extracted at approximately bit 6 or less. Conversely, the error probability for the response value increases as the selected bit position approaches bit 0. This means that the probability of obtaining the same response value for the same challenge is reduced, resulting in poor reliability characteristics. Several experiments have shown that bit 5 is the most appropriate bit position given the uniqueness and reliability of the implemented RC PUF board, although bit 4 to bit 6 can be used as response bits. Once the bit position is selected, the same bit position can be used for the same type of device. However, if the design changes, it is necessary to reselect the appropriate bit position considering several factors, such as values, types, and tolerances of RC components.

Since we fixed the ADC bit position, it is possible to evaluate the performance of the RC PUF. In order to characterize the RC PUF, we use three commonly employed performance metrics: uniqueness, reliability (also known as steadiness), and randomness [1,17,28].

*Uniqueness* represents the variations in the responses of multiple devices to the same challenge and can be referenced as extra-chip variation (EC), which is calculated as [1,17,29]

(12) $$EC=\frac{1}{M\left(M-1\right)N}\overset{M}{\underset{i=1}{\sum }}\overset{M}{\underset{k=1,k\neq i}{\sum }}\overset{N}{\underset{j=1}{\sum }}\frac{HD\left(r_{i,j},\bar{r_{k}}\right)}{n}\times 100\%,$$

where *M* is the number of devices, *n* is the length of response, *N* is the number of *n* bit responses per device, $r_{i,j}$ is the *j*-th response sample from the *i*-th board, $\bar{r_{k}}$ is the mean value of the *N* responses from the *k*-th board, and *HD* represents the hamming distance. We note that the optimal value for uniqueness is 50%

*Reliability* or *steadiness* represents the ability of a particular device to generate the same response and can be referenced as intra-chip variation (IC), which is given by [1,17]

(13) $$IC_{i}\left(T,V\right)=\frac{1}{N}\overset{N}{\underset{j=1}{\sum }}\frac{HD\left(r_{i,j},r_{i,ref}\right)}{n}\times 100\%,$$

where $r_{i,ref}$ is the mean value of responses of the *i*-th board. We note that this metric depends on the environmental parameters, such as temperature (*T*) and voltage (*V*). The percentage figure for reliability can be defined as

(14) $$Reliability=100-IC_{i}\left(T,V\right).$$

Obviously, the optimal value for reliability is 100%.

For the randomness test, we use a subset of NIST randomness test suit (NIST SP 800-22) [30]. To evaluate the performance of RC PUF, a total of 100 RC PUF boards were tested for the above three metrics.

In addition to the performance metrics, we also describe the characteristics of the RC PUF for input challenges with similar bit patterns or hamming weights.

### 3.1. Uniqueness and Reliability

For the uniqueness and reliability test, the evaluation of the RC PUF consisting of two different RC circuits, RC1 and RC2, was performed with 32,768 random challenges for each RC PUF, where each 32-bit challenge was repeated 100 times. Then majority voting was applied to determine the response bit. In order to improve the reliability of the experiment, 1-bit response was obtained by repeating 100 times for one challenge. However, since the error correction method, such as a fuzzy extractor, may be used in the actual execution environment, it will be enough to repeat 1~3 times considering the reliability characteristics of RC PUF.

The MCU used in this experiment operates at 96 MHz, but the time required to obtain 1-bit response is determined by the bit delay time, not the MCU’s operating speed. For example, if we set the bit delay time to 2 us in FD mode and repeat 3 times for the same 32-bit challenge to generate the 1-bit response, it takes approximately 192 us to generate one response. In VD mode, each MCU uses its own bit delay time, resulting in different response generation times.

We first tested the RC PUF with the bit delay time fixed (FD) at 2 us and 32 us to evaluate the performance change over the bit delay time. Next, we added 4-bit and 5-bit MCU-specific temperature sensor calibration data to the initial bit delay time so that the newly generated bit delay times were between 8~24 us (VD4) and 1 ~ 32 us (VD5), respectively. For example, if we run RC PUF in FD mode, then the same bit delay time ($T_{bd}$) is used for all challenges in all RC PUF boards. However, for VD4 or VD5 modes, $T_{bd}$ is unique among different RC PUF boards but the same for all challenges in one RC PUF board. The procedure for setting the bit delay time is the same as described in [17].

Table 1 shows the uniqueness and reliability performance in FD and VD modes for both RC1 and RC2. The performance values for RC1 are the same as described in [17], and new performance values for RC2 are added. As shown in Table 1, when the fixed bit delay time (FD) increases from 2 us to 32 us, the uniqueness and reliability are improved in both RC1 and RC2. This is because the longer the operating time is, the more differences are accumulated due to the tolerance of the RC circuit. When using the MCU-specific bit delay time (VD4 and VD5), both types of RC circuits have more than 48% uniqueness, which is close to the ideal value of 50%, while achieving more than 98% reliability.

In several experiments, we can see that the difference in ADC values between different PUFs is in the range of several tens to hundreds when using different bit delay times, i.e., VD4 and VD5, resulting in a better uniqueness than using the same fixed delay time for all PUFs. These results can be observed from the standard deviation of the difference in ADC values for the same challenge when using the variable bit delay time, which will be discussed later in Section 3.4.

Since the RC PUF uses the response of sequential unit step inputs to the RC circuit, the output of the RC circuit can be represented by a series of an exponential curve that steeply rises or falls at the beginning of the input and then converges to a constant value as the slope of the curve decreases over time as shown in Equations (1), (2), or (9). As explained in Section 2.1, the generation and maintenance of the bit delay time of the RC PUF and the latching of the ADC value are done by the MCU. If the bit delay time is small to use only the initial stage of the exponential curve, the error rate increases due to the relationship between the accuracy of the MCU clock and the fast change rate of the exponential curve.

For example, the time constant for RC1 is calculated as *τ* = 20 us for *R* = 100 kΩ and *C* = 200 pF, although the measured time constant is larger than this by the parasitic components on the board. If the bit delay time is fixed to 2 us, corresponding to 1/20 of the calculated time constant, the output of RC1 changes at the initial stage of the exponential curve. In this case, because the output curve of RC1 changes faster than RC2, the reliability of RC1 is 1.5% lower than that of RC2 as shown in Table 1. On the other hand, if the bit delay time is large enough to be 32 us, the reliability of RC1 and RC2 is equal to 98.5%. In VD4 and VD5 modes, because the small and large bit delay times are mixed, the reliability values are similar for RC1 and RC2 with a small difference of 0.1% ~ 0.2%. Since we only used 100 boards in the experiment, it is necessary to test on even more boards to distinguish these sub-dot differences.

**Figure 6:**
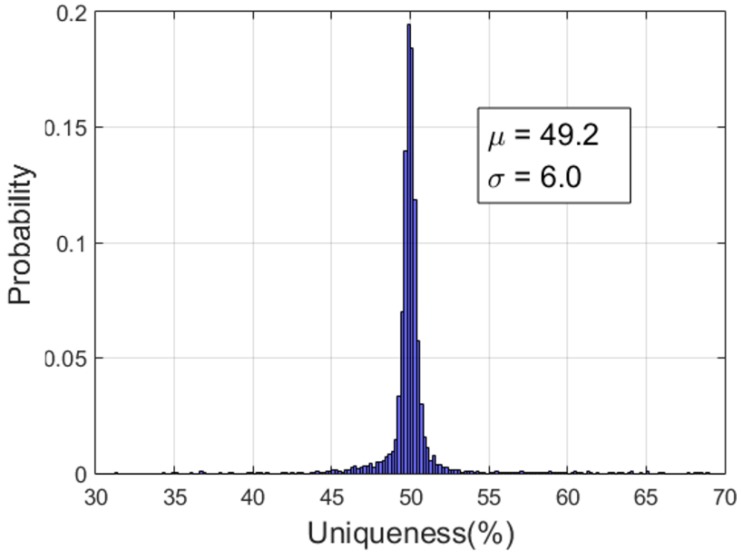
Uniqueness for RC2-VD5.

The bit delay time also affects the uniqueness characteristics of the RC PUF. In the fixed mode, when the bit delay time is 2 us, the uniqueness value of RC2 is 1.5% higher than that of RC1, but when 32 us, the uniqueness value of RC1 and RC2 becomes similar within 0.2%. In VD4 and VD5 modes, the uniqueness values of RC2 are 0.3% to 0.4% higher than those of RC1. We think that because RC2 uses more RC components than RC1, it is more affected by the accumulation of tolerance values of these components and the parasitic resistance and capacitance on the board. Figure 6 shows the uniqueness characteristic of RC2 in VD5 mode.

### 3.2. Randomness

In contrast with the uniqueness metric, there is no specific formula measuring the randomness of PUFs. A commonly used method is measuring the bias of the PUF response, but this is not sufficient to represent the randomness of PUFs [1]. For this reason, some previous works have employed the NIST SP 800-22, originally designed to evaluate the performance of the random number generator (RNG), to measure the randomness of PUFs [1,8,14].

To evaluate the randomness of the RC PUF, we selected nine subsets of the NIST statistical test suite because some test cases of the NIST SP 800-22 must be performed using a minimum stream size of 10^6^ bits, which is generally not suitable for a PUF [1]. To better understand how the nine test suites are used, each one is briefly described as follows.

(1) Frequency Test (T1): The first test measures the proportion of 0′s and 1′s for the binary sequence.
(2) Frequency Test within a Block (T2): The second test is similar to the first Frequency Test but measures the number of 1′s within M-bit blocks.
(3) Cumulative Sums Test (T3): The purpose of this test is to measure the maximal excursion of the binary string. In other words, the initial value of the Cumulative Sums is set to 0 and add or subtract to this value for each 1 and 0 of the binary sequence. The final value of the Cumulative Sums shows the random walk characteristics of the binary sequence.
(4) Runs Test (T4): This test aims to evaluate linear dependencies between fix-length of bit strings inside the binary sequences.
(5) Longest Runs Test (T5): This test checks the irregularity of the longest run of 1′s within M-bit subblocks of the binary sequences.
(6) Rank Test (T6): The focus of this test is to measure the linear dependencies among fixed length substrings of the binary sequences.
(7) Discrete Fourier Transform (DFT) Test (T7): The purpose of this test is to calculate the peak value through the DFT and use it to detect repetitive patterns near each other within the binary sequence.
(8) Approximate Entropy Test (T11): This test focuses on the frequency of all possible fixed-length of consecutive overlapping patterns in the binary sequences.
(9) Serial Test (T14): The last test aims to check the frequency of every overlapping m-bit pattern across entire binary sequences.

**Figure 7:**
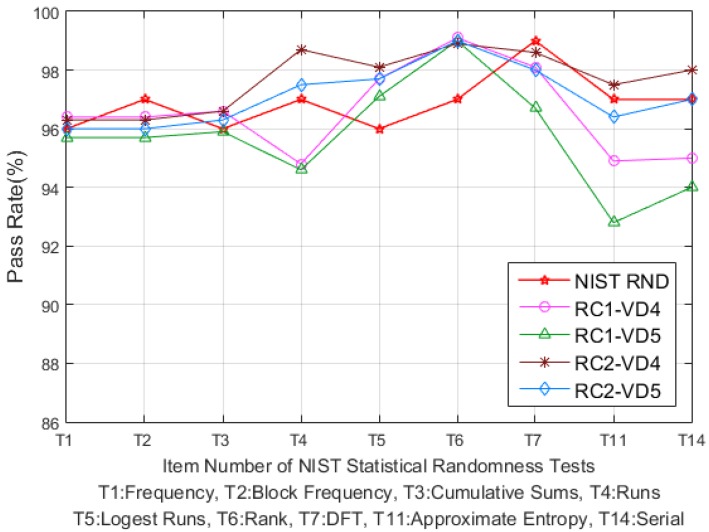
Results of randomness test for RC PUF using NIST test suite.

For the selected subset, we performed the test using VD4 and VD5 bit delays for both RC1 and RC2, and the 1,048,576 response bits were generated using randomly selected challenges for each of the 100 RC PUFs. The results of all test suites were tested using the response of each RC PUF, and the final results were expressed as the average of 100 RC PUFs. As shown in Figure 7, the pass rates for all combinations are higher than 90%, and RC2 combinations of RC2-VD4 and RC2-VD5 show similar pass rates compared to the NIST recommended random data (NIST RND) [30]. Any bit bias for the entire bit strings of the PUF output can be checked using T1, T2 or T3 [11], which exhibits pass rates >0.95 for all combinations. However, the result of T4 shows that RC1 has more consecutive 1′s or 0′s than RC2. In addition, T11 and T14 show that RC2 has better irregularity characteristics for overlapping patterns than RC1. More specifically, two RC PUFs showed below 15% pass rate in T11 for VD5 mode in RC1. Their response includes bit strings with similar bit patterns, such as “…1100000000110000…” or “…0011000110001100…”, resulting in relatively low pass rates. In addition, since one response bit is generated from one random challenge, it is difficult to physically explain why similar bit patterns are generated, and it is unclear whether it is caused by structural differences between RC1 and RC2. Nevertheless, the results of all NIST test suites for the RC PUF in Figure 7 show that RC2 has better randomness characteristics than RC1, and further analysis is required using more samples.

### 3.3. Stability against Voltage and Temperature Variations

We consider two environmental variations such as temperature and voltage to evaluate the stability of the RC PUF. The evaluation was performed with a 1024-bit reference response over 10 randomly selected RC PUF boards under normal conditions such as room temperature of 25 °C and supply voltage of 3.3 V, and FD, VD4, and VD5 bit delay cases are considered. Then the stability was calculated based on how many bit flips occurred compared with the reference response.

**Figure 8:**
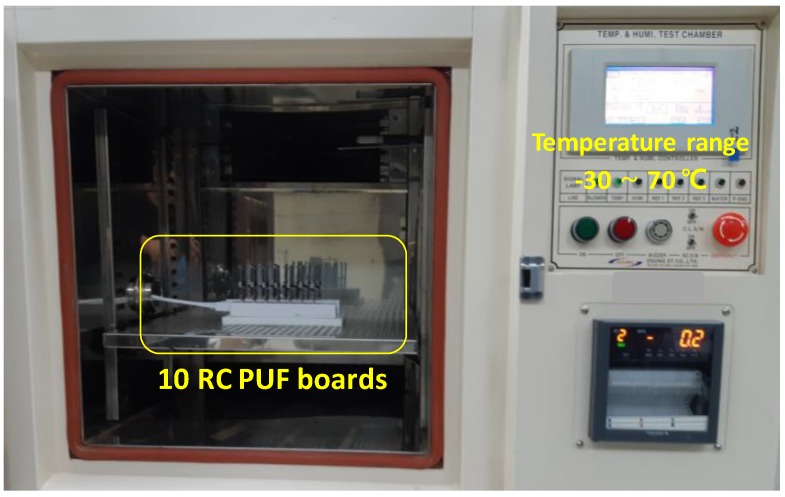
Temperature chamber test environment.

**Figure 9:**
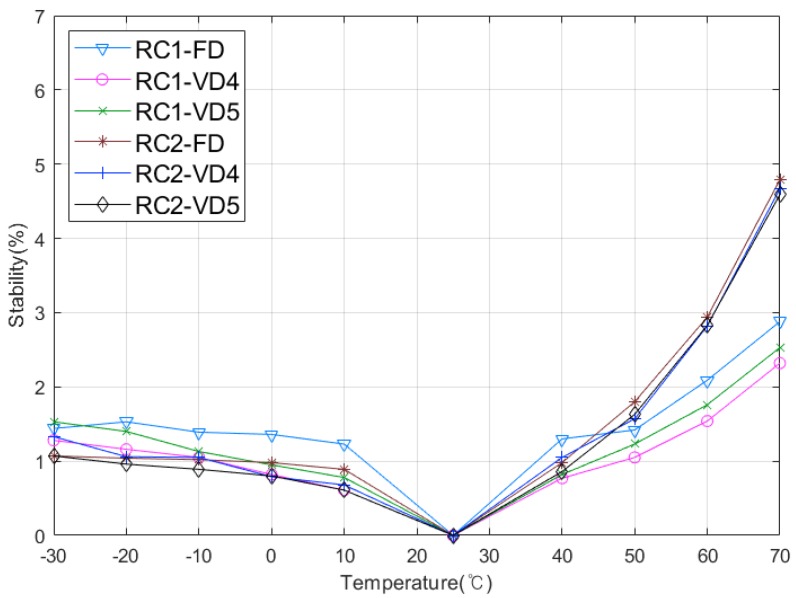
Stability over temperature variation.

First, to evaluate the stability to temperature variations, the RC PUF boards were placed on a temperature chamber, as shown in Figure 8, and the temperature was increased by 10 °C from –30 °C to 70 °C. Figure 9 shows that the stabilities of both RC1 and RC2 are maintained within 2% at temperatures below 50 °C, but increases above 50 °C, as shown in Figure 9. More specifically, RC1 exhibits less than 3% stability over the entire range, but the stability of RC2 increases by 5% above 50 °C. We think that this difference is due to the number of resistors used in the RC circuits. As explained earlier, we exploit class 1 (C0G) capacitors, which are robust to temperature variation and aging, while the resistors used in RC circuits are commonly used thin film resistors with slightly different characteristics over temperature changes. Two thin film resistors are used in RC2, increasing the error rate at higher temperatures. Although the stability increases above 50 °C, the worst case 5% stability on the RC2 is an acceptable error rate that can be recovered using commonly used error correction codes [6].

**Figure 10:**
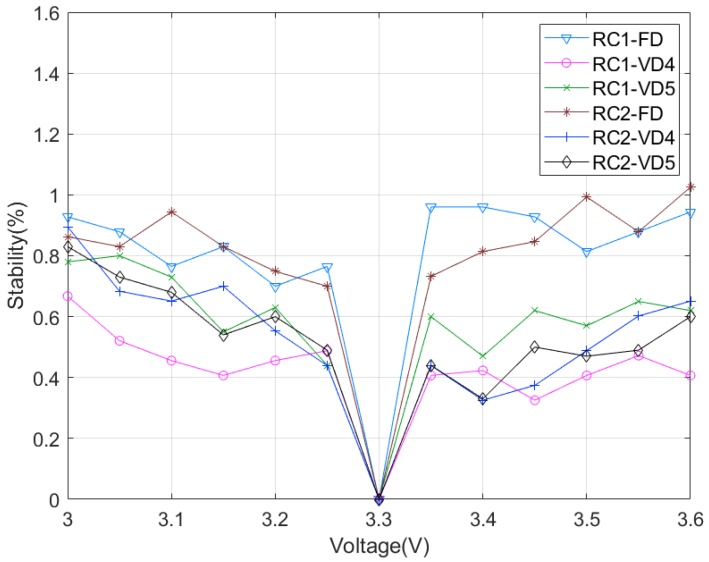
Stability over voltage variation.

Next, the stability to voltage changes was measured in 0.05 V steps over a voltage range of 3.0~3.6 V. Figure 10 reveals less than 1% stability over the entire range for both RC1 and RC2, which corresponds to a bit flip of less than 10 bits of 1024 bits. The main reason that the RC PUF is robust to voltage variations is because the power supply voltage of the MCU is equal to both the output voltage of the GPIO used as the input voltage of the RC circuit and the operating voltage of the ADC. Therefore, the ADC value is determined by the voltage change ratio, not the absolute voltage.

### 3.4. RC PUF Behavior against Various Challenge Bit Patterns

In the RC PUF, the final output value of the RC circuit is determined by the input challenge. Therefore, it is important to look at the different behavior of the RC PUF for input challenges with similar bit patterns or hamming weights. As shown in Table 2, we first tested the RC PUF by fixing the bit delay times (FD) 8 us for RC1 and 16 us for RC2. We also used 5-bit MCU-specific temperature sensor calibration data, which has been explained in Section 3.1, to generate variable bit delay times (VD4 and VD5). Then we compared the average and standard deviation of the final latched ADC values for each RC1 and RC2 over 10 randomly selected RC PUF boards.

In order to make the reference ADC values, we first run the RC PUF with the initial 32-bit challenge of 0x5A5A5A5A, where the least significant bit (LSB) of bit 0 is the first bit of the input sequence and the most significant bit (MSB) of bit 31 is the last input to the RC circuits. Therefore, the input sequence for the initial input challenge becomes 01011010…01011010 and the number of 0’s and 1’s in the entire input sequence is the same.

Next, we apply different values as input challenges such as 0xA5A5A5A5 and 0xCCCCCCCC, which have the same number of 0’s and 1’s but different bit patterns compared to the initial challenge. As shown in Table 2, it can be seen that there are significant differences in ADC values for input challenges with different bit patterns compared to the ADC value generated by the initial challenge in FD, VD4, and VD5 mode for both RC1 and RC2. This result shows that although input challenges consist of the same number of 0′s and 1′s, it is difficult to guess the ADC value according to input challenges with different bit patterns.

In fact, we note that the latched ADC value is affected by the bit pattern closer to the final bit in the input sequence, rather than the number of 0’s and 1’s and the overall bit pattern. For example, when 0x5ACCCCCC is used as the input challenge, only the last 8 bits of the input sequence are the same as compared to the initial challenge. In this case, it can be seen that there are significant differences in FD mode between the ADC value generated by the initial challenge and the ADC value generated by 0x5ACCCCCC as shown in Table 2.

However, when 0x5A5CCCCC is used as an input challenge so that the last 16 bits are the same as the initial challenge, the latched ADC value is almost similar to the ADC value generated by the initial challenge in FD mode. Furthermore, the standard deviation in the FD mode shows that the ADC values are very similar on all RC PUF boards. This result can be one of the reasons that the uniqueness in FD mode is worse than those of VD4 and VD5 modes.

In VD mode, the standard deviations are larger than those in FD mode because the bit delay times on each board are different. The ADC values for the 0xA5A5A5A5 and 0xCCCCCCCC challenges differ significantly from the ADC value of the initial challenge, as in FD mode. In VD5 mode, the ADC values for input challenge of 0x5ACCCCCC and 0x5A5CCCCC in RC1 and RC2 are significantly different from the ADC values for the initial challenge, but in VD4 mode, RC2 shows similar values to the ADC values of the initial challenge for the previous input challenge. However, because the ADC value for RC2 in VD4 mode is the average value and the standard deviation is greater than 70, it can be thought that the ADC values are evenly distributed.

Next, the differences in ADC values between the initial challenge of 0x5A5A5A5A and 0x5A5CCCCC are 63 in RC1, 1 in RC2 for VD4, and 86 in RC1 and 10 in RC2 for VD5 mode, which is greater than in FD mode. As expected, since the deviation of the bit delay time in VD5 is greater than VD4, the standard deviation of latched ADC values of VD5 is greater than VD4, with some exceptions.

As a result, VD4 and VD5 mode show less similarity of the latched ADC values according to the bit pattern of input sequences than FD mode, and VD5 mode is less affected by the bit pattern close to the last bit in the input sequence than VD4. Although the uniqueness of the RC PUF can be affected by the similarity of the bit patterns closest to the last bit rather than the number of 0’s and 1’s of the input sequence, the standard deviation of VD4 and VD5 mode is large enough to ensure uncorrelated responses to the same input challenge.

## 4. Conclusions

In this paper, we presented a novel RC PUF that can be easily implemented using one MCU with ADC function and several low-cost passive components such as resistors and capacitors. The structure of the proposed RC PUF is very simple and easy to implement in resource-constrained IoT devices. Experimental results showed that the proposed RC PUF achieved 49% uniqueness and 98% reliability by appropriately selecting those components and using MCU-specific helper data. Thanks to the structural characteristics, the RC PUF showed less than 1% stability over 10% voltage variation.

We characterized the RC PUF with two different RC circuits. Comparing the experimental results of the two RC circuits, both types showed similar reliability and stability over voltage variation, while the second-order RC circuit had slightly better uniqueness and randomness. On the other hand, the first-order RC circuit showed better stability against temperature variation, although the stability of the second-order RC circuit at high temperature was below 5%. In summary, the second-order RC circuit is suitable for the RC PUF for use in typical applications, and the first-order RC circuit can also be considered for products where implementation cost is important.

Future works will include probing analysis against the proposed RC PUF with various types of RC circuits and operating conditions. Other passive components will also be considered to make PUF-like characteristics. A generalized randomness test using more samples will also be included in our future works.

## Figures and Tables

**Table 1:** Uniqueness and Reliability of the RC PUF.

RC Type	Metric	Delay Mode (Bit Delay Time)			
		FD (2 us)	FD (32 us)	VD4 (8~24 us)	VD5 (1~32 us)
RC1	Uniqueness (%)	27.3	30.9	48.5	48.8
	Reliability (%)	96.2	98.5	98.2	98.3
RC2	Uniqueness (%)	28.7	30.7	48.8	49.2
	Reliability (%)	97.7	98.5	98.1	98.1

**Table 2:** Latched ADC values and standard deviations of the RC PUF with various input challenges.

Challenge	RC Type	Delay Mode (Bit Delay Time)					
		FD		VD4		VD5	
		ADC Value	Standard Deviation	ADC Value	Standard Deviation	ADC Value	Standard Deviation
0x5A5A5A5A	RC1	1692	1.03	1213	86.3	1524	104.5
	RC2	1777	1.87	1430	74.3	1998	74.0
0xA5A5A5A5	RC1	2407	1.55	2816	90.5	2490	110.1
	RC2	2320	2.56	2667	74.6	2102	73.3
0xCCCCCCCC	RC1	2869	0.75	3412	100.3	2992	157.1
	RC2	2956	3.72	3380	80.4	2576	147.9
0x5ACCCCCC	RC1	1736	0.98	1284	91.9	1638	125.5
	RC2	1798	2.64	1432	75.4	2062	93.9
0x5A5CCCCC	RC1	1697	0.54	1276	83.1	1610	113.4
	RC2	1778	2.31	1429	73.5	2008	79.4
